# Using Fish Skin Gelatin Hydrolysate as Stabilizer and/or Emulsifier Agent in Ice Cream Production and Melting, Textural, Rheological, and Sensory Characteristics

**DOI:** 10.3390/gels11080643

**Published:** 2025-08-14

**Authors:** Sefik Tekle, Hamza Goktas, Cansu Agan, Aysen Develioglu-Arslan, Zeynep Hazal Tekin-Cakmak

**Affiliations:** 1 Food Engineering Department, Faculty of Engineering and Architecture, Kirsehir Ahi Evran University, Kirsehir 40100, Turkey; 2 Food Engineering Department, Engineering Faculty, Bolu Abant Izzet Baysal University, Bolu 14030, Turkey; 3 Food Technology Program, Vocational School, Istinye University, Istanbul 34010, Turkey; cagan@istinye.edu.tr; 4 Culinary Program, Vocational School, Istinye University, Istanbul 34010, Turkey; aysen.arslan@istinye.edu.tr; 5 Food Engineering Department, Chemical and Metallurgical Faculty, Yildiz Technical University, Istanbul 34210, Turkey; hazal.cakmak@yildiz.edu.tr

**Keywords:** fish skin gelatin hydrolysate, stabilizer, emulsifier, ice cream, rheological properties, overrun levels, melting properties, textural properties, sensory properties

## Abstract

The increasing global interest in fish consumption leads to a greater generation of fish waste. Fish waste, rich in nutrients such as protein, bioactive compounds, and vitamins, is attracting growing attention for its potential applications in food. In this study, gelatin hydrolysate obtained from fish skin waste was utilized as a stabilizer and/or emulsifier in ice cream production. It was found that gelatin hydrolysate significantly increased the protein content of the ice cream samples. The *K* and *n* values in different ice cream compositions varied between 0.009 and 1.012 Pa.s^*n*^ and 0.356 and 0.863, respectively. The consistency coefficients of samples D1 (sahlep and mono-diglyceride) and D3 (sahlep and gelatin hydrolysate) were almost the same, indicating that the mono-diglyceride was replaced by an equivalent amount of gelatin hydrolysate. All the ice cream mixtures tested showed non-Newtonian, pseudoplastic flow, as indicated by their *n* values being less than 1. All mixtures demonstrated greater elasticity than viscosity, as their storage modulus (*G*′) was higher than their loss modulus (*G*″). In the third interval of 3-ITT, all ice cream mixtures displayed thixotropic behavior, indicating that their viscoelastic properties could be restored after a sudden deformation. The overrun levels of the samples ranged from 9.55% to 21.74%; the use of gelatin hydrolysate resulted in a statistically significant increase (*p* < 0.05). The highest hardness and stickiness values in the samples were determined in the specific sample containing equal amounts of emulsifier, stabilizer, and gelatin hydrolysate. Furthermore, gelatin hydrolysate prolonged the first dripping time and melting rate of the samples.

## 1. Introduction

The global consumption of fish is on the rise, with estimates suggesting that approximately 60% of fish waste is generated during the processing of fish products [1]. Fish waste is an excellent source of high-quality compounds suitable for human consumption, and it is rich in protein and contains a variety of bioactive and nutritionally valuable macronutrients and micronutrients, including antioxidants, enzymes, and bioactive peptides [1,2]. Additionally, fish waste products are an excellent source of protein [2]. It is stated that the fundamental protein composition of fish waste ranges from 8% to 35%. Therefore, fish proteins are significant candidates for utilization in pharmaceuticals and functional food production [3]. It is possible to obtain protein hydrolysate, gelatin hydrolysate, collagen hydrolysate, and bioactive peptides from fish waste. Owing to the favorable foaming and emulsifying characteristics of fish gelatin hydrolysates, they can be employed as emulsifiers and emulsion-stabilizing components in a range of products [4].

Ice cream is a frozen dairy product made of an aerated emulsion (O/W) consisting of partially fused fat globules, air bubbles, and ice crystals [5]. It is enjoyed not only for its nutritional benefits but also for its cooling effect during summer [6]. Ice cream is made with milk, cream, and sugar, but its quality especially depends on the emulsifiers and stabilizers used [7]. While emulsifiers mostly utilized in ice cream are mono-diglycerides, lecithin, and polysorbates, stabilizers are guar gum, carboxymethyl cellulose, xanthan gum, and sahlep. Emulsifiers serve to mitigate the development of ice crystals by enhancing the volume increase capacity and resistance to melting, imparting a dry and hard texture, and providing a smoother consistency and oily sensation [8]. The initial objective of utilizing stabilizers in ice cream production is to achieve a uniform structure and texture, to minimize the formation of ice crystals, particularly in response to temperature fluctuations during storage, and to enhance the product’s resistance to melting [9]. Additionally, stabilizers are favored in ice cream production because they play vital roles, such as increasing viscosity and smoothness of the mix, enhancing aeration, and slowing down structural collapse during melting [5].

Fish processing waste has increased in recent years, creating major challenges for environmental sustainability and economic value addition [2]. Additionally, the food industry is increasingly interested in using natural, functional, and sustainable ingredients in food formulations [10]. A review of the literature reveals a paucity of studies examining the utilization of gelatin hydrolysate in ice cream production. Nevertheless, the effect of soy protein hydrolysate on melting characteristics of ice cream and its potential use on low-fat ice cream production have been investigated in a number of studies [11,12,13]. Additionally, López-Martínez et al. [14] developed an ice cream product utilizing egg white hydrolysate and conducted an investigation into its sensory properties. In this context, no studies have been identified in the literature on using fish skin gelatin hydrolysate as a stabilizer or emulsifier in ice cream production. This study aims to explore the potential application of gelatin hydrolysate derived from fish skin waste as a traditional stabilizer or emulsifier in ice cream, thereby supporting waste management and providing a functional ingredient for food formulations. The physicochemical, textural, melting, rheological, and sensory properties of ice cream samples containing fish skin gelatin hydrolysate were analyzed.

## 2. Results and Discussion

### 2.1. Physicochemical and Color Properties

The pH and titratable acidity (TA) values of the samples ranged from 6.45 to 6.48 and from 2.69 to 2.83 (%), respectively (Table 1). These findings showed that the utilization of gelatin hydrolysate as an emulsifier and/or stabilizer did not significantly change the acidity values of the ice creams (*p* > 0.05). In a similar manner, the total dry matter (TDM) values of the samples ranged from 29.84 to 30.04 g/100 g, and the utilization of gelatin hydrolysate did not exert a significant effect on the TDM value of the ice creams (*p* > 0.05). Conversely, the utilization of gelatin hydrolysate as an emulsifier and/or stabilizer resulted in a substantial increase in the protein content of the ice creams (*p* ≤ 0.05). In this context, the protein content of the ice creams with a high amount of gelatin hydrolysate was high. Therefore, the highest protein content was found in sample D4 (gelatin hydrolysate) with 3.99 g/100 g, whereas the lowest protein content was found in sample D1 (sahlep and mono-diglyceride) without gelatin hydrolysate. The utilization of gelatin hydrolysate did not significantly affect the brightness value (*L⃰*) of the ice cream samples, and no significant discrepancy was observed between the samples (*p* > 0.05) (Table 1). The utilization of gelatin hydrolysate resulted in variations in the *a⃰* values of the ice creams. Consequently, a significant decrease was detected in the *a⃰* values of ice cream samples containing gelatin hydrolysate, with the exception of sample D3 (*p* ≤ 0.05). The highest *a⃰* value was detected for sample D2 (mono-diglyceride and gelatin hydrolysate). In the *b⃰* values of ice creams, a significant difference was detected only between samples D2 (mono-diglyceride and gelatin hydrolysate) and D3 (sahlep and gelatin hydrolysate) regarding the use of gelatin hydrolysate (*p* ≤ 0.05).

### 2.2. Overrun Levels

**Figure 1:**
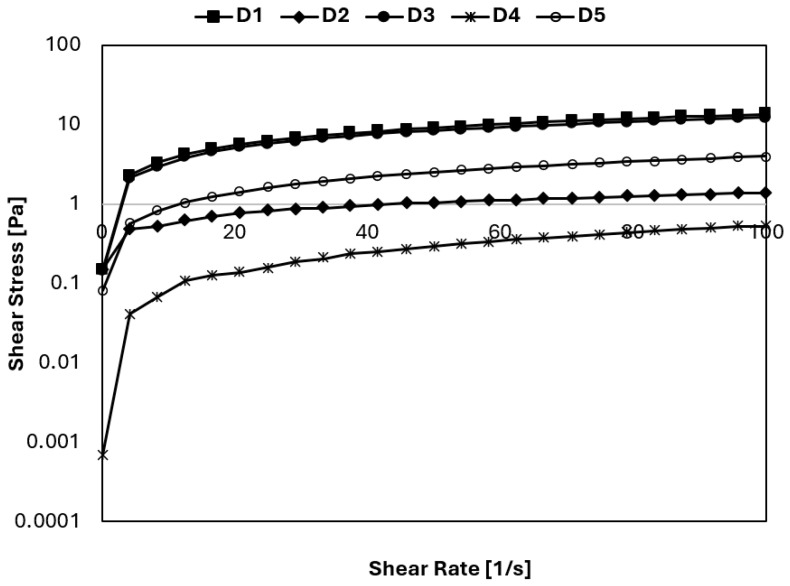
Steady shear rheological behavior of the ice cream mixes.

The overrun is used to denote the rate of air mixing into the ice cream, a process which is of significance for both the texture and the sensory properties of the ice cream. The overrun values of ice cream samples varied between 9.55% and 21.74% for D2 (mono-diglyceride and gelatin hydrolysate) and D3 (sahlep and gelatin hydrolysate) samples, respectively (Table 1). Statistically significant differences for overrun levels of ice cream samples were determined (*p* ≤ 0.05). In general, the use of gelatin hydrolysate in ice cream production resulted in an increase in overrun levels. Thus, the highest overrun level was detected in the D3 (sahlep and gelatin hydrolysate) ice cream sample containing sahlep and gelatin hydrolysate in combination. Conversely, the lowest overrun level was detected in sample D2 (mono-diglyceride and gelatin hydrolysate), which contained mono-diglyceride and gelatin hydrolysate; no significant difference was observed in comparison to the control sample. The overrun level of sample D4 (gelatin hydrolysate), which contained only gelatin hydrolysate, was higher than the control sample (*p* ≤ 0.05). The findings of this study have demonstrated that the use of gelatin hydrolysate in ice cream production resulted in an increase in overrun levels. However, the combination of sahlep and gelatin hydrolysate resulted in an increase overrun, while the combination of mono-diglyceride and gelatin hydrolysate resulted in a partial decrease in overrun ice cream samples. Consistent with this study, Liu et al. [12] and Yan et al. [13] reported observing an increase in the overrun level of ice cream when soy protein hydrolysate was used as a fat replacer. Rheology results showed that the viscosity of the ice cream samples decreased with increasing shear rate. The low overrun level observed in sample D2 may be due to the viscosity of the ice cream sample. It has been reported that a sample needs a certain viscosity to achieve a moderate level of overrun [12]. However, adding gelatin hydrolysate did not increase viscosity enough to create a viscous structure that would help with air entrainment and raise the overrun level. One factor affecting the overrun level of ice cream is foaming capacity. Fish skin gelatin hydrolysate is known to have high foaming capacity [15]. The high overrun levels of ice cream samples D3, D4, and D5 containing gelatin hydrolysate may be related to foaming from the gelatin hydrolysate. The addition of gelatin hydrolysate did not cause an increase in the viscosity of the ice cream (D4 ice cream sample, Figure 1), resulting in a less viscous structure that may help in air incorporation and result in an increase in the overrun [12].

### 2.3. Textural Characteristics of Ice Cream Samples

The highest hardness and adhesiveness values (2826.95 g, −606.35) were determined in the D5 (sahlep, mono-diglyceride, and gelatin hydrolysate) sample, while the lowest hardness and adhesiveness values (803.19 g, −92.05) were determined in the D4 (gelatin hydrolysate) sample. High hardness indicates that the ice cream has a denser and more resistant structure, while low hardness may indicate a soft and easily meltable structure. However, high adhesiveness values may cause the ice cream to have a stickier feeling in the mouth. Both the hardness and adhesiveness values of the ice cream sample (D4), which contained only gelatin hydrolysate as an emulsifier and stabilizer, were found to be significantly lower (*p* ≤ 0.05). However, the use of sahlep, mono-diglyceride and gelatin hydrolysate in equal proportions resulted in a harder and stickier ice cream. On the other hand, a slight increase in hardness was observed in ice cream samples containing emulsifier and gelatin hydrolysate (D2 sample), and sahlep and gelatin hydrolysate (D3 sample), compared to the control, while a slight increase in adhesiveness was seen only in the ice cream sample containing emulsifier and gelatin hydrolysate (D2 sample) (*p* > 0.05). Factors that impact ice cream hardness include solids content, mix viscosity, bubble size, expansion rate, and distribution of ingredients. However, the main factor is the overrun level of the ice cream with higher overrun levels expected to result in a softer texture [13]. Consistent with reports in the literature, a relationship was observed between the overrun and hardness values of ice cream samples D2 (mono-diglyceride and gelatin hydrolysate) and D4 (gelatin hydrolysate). For sample D2, hardness increased as the overrun level decreased compared to the control sample, whereas for sample D4, hardness decreased as the overrun level increased. Another factor affecting the hardness of ice cream is the viscosity of the mix [16,17]. The lower viscosity of sample D2 resulted in higher hardness [13]. Conversely, when both overrun and viscosity values were considered for ice cream samples D3 (salep and gelatin hydrolysate) and D5 (equal proportions of salep, mono-diglyceride, and gelatin hydrolysate), no similar relationship with hardness values was found. This may be related to the varying concentrations of salep, emulsifier, and gelatin hydrolysate in the ice cream mixes.

### 2.4. Melting Characteristics of Samples

The first dripping time (FDT) of the ice creams varied between 3.74 ± 0.56 and 6.67 ± 0.63 min (Table 2). The long FDT indicates that the ice cream is more resistant to melting. The D4 (gelatin hydrolysate) ice cream sample was more resistant to melting than other ice cream samples. D1 (sahlep and mono-diglyceride) ice cream sample had the shortest first dripping time of 3.74 min. The high melting rate (MR) indicates that the ice cream melts more quickly, and the D1 ice cream sample has the highest melting rate of 0.62 (g/min). Therefore, the melting of the D1 ice cream sample occurred more quickly than that of other ice creams containing gelatin hydrolysate. Finally, the use of gelatin hydrolysate in ice cream production may have led to a delay in melting because it affected the overrun level of the ice cream. The rate at which ice cream melts is linked to its overrun level, with ice creams having lower overrun levels melting at a faster rate because air cells in ice cream act as a barrier against heat transfer [12,16]. High overrun levels confirmed that D3, D4, and D5 ice cream samples containing gelatin hydrolysate melted more slowly compared to the control. In many studies, the melting of ice cream has been linked to the overrun level, with rapid or delayed melting associated with low or high overrun levels, respectively [11,12,13,16]. On the other hand, melting of ice cream is influenced by factors beyond overrun level, such as the type and amount of stabilizers and emulsifiers, as well as its composition [18]. In this respect, the slower melting of sample D2, despite its low overrun level, may be related to its composition.

### 2.5. Flow Behavior and Rheological Properties

The flow behavior and rheological properties of ice cream samples are closely linked to their technical features. Ice cream usually shows pseudoplastic (shear-thinning) behavior, meaning its viscosity drops as shear rate rises [7]. This behavior eases processing steps like mixing and pumping and also gives a creamy, rich mouthfeel for consumers [6]. High viscosity and yield stress help maintain shape and slow melting, improving shelf life and structural stability [19,20]. Additionally, the dominance of elastic properties (*G*′) over viscous ones (*G*″) supports the stability of the air structure (overrun), preventing collapse and ensuring a light, foamy texture [20,21]. These rheological characteristics directly affect key technical qualities of ice cream, such as consistency, spreadability, melting behavior, and overall sensory appeal [20,21]. Therefore, optimizing flow behavior and rheological features in ice cream formulations is crucial for both processing efficiency and consumer satisfaction.

As seen in Figure 1, the gradient of the shear stress versus shear rate curves of the mixes decreased, suggesting that all mixes demonstrated a reduction in viscosity as the shear rate increased. The structural breakdown of the intermolecular interaction explains the decrease in viscosity [22]. The ice cream mixes showed shear-thinning flow characteristics, a typical behavior for an ice cream mix. The shear-thinning flow directly impacts the mixing process and is important in selecting the pump size [22].

The steady shear rheological parameters (*K* and *n*) were described with the Power Law model. The determination coefficient (R^2^) was also calculated for ice cream mixes and shown in Table 2. Table 2 summarizes the effects of different amounts of stabilizer, emulsifier, and gelatin hydrolysate on ice cream mixture production. R^2^ values of the Power Law model were higher than 0.989. The accuracy of this model fitting was high, as indicated by high correlation coefficients. K and *n* values of the mixes were measured as 0.009–1.012 Pa.s*^n^* and 0.356–0.863, respectively. The *n* values of ice cream mixes were less than 1; thus, the mixes exhibited pseudoplastic flow (Table 2), and dairy products are generally characterized by pseudoplastic flow [23]. As seen in Table 2, the *K* values of sample D3, containing 0.75 g stabilizer per 100 mL milk and 0.25 g gelatin hydrolysate per 100 mL milk, and sample D1, containing 0.75 g stabilizer per 100 mL milk and 0.25 g emulsifier per 100 mL milk, are very close to each other. Evaluating this result, it can be concluded that when the amount of stabilizer (0.75 g/100 mL milk) is kept constant, the use of gelatin hydrolysate instead of the same amount of emulsifier affects consistency similarly. This change could be attributed to the hydration of protein and stabilizers during aging, thereby changing viscosity. However, it can be seen from Table 2 that the amount of stabilizer plays an important role in the consistency of ice cream samples. While the *K* value of sample D1 (0.75 g stabilizer/100 mL milk and 0.25 g emulsifier/100 mL milk) is 1.012 Pa.s*^n^*, the *K* value of sample D2 (0.75 g gelatin hydrolysate and 0.25 g emulsifier) is 0.249 Pa.s*^n^*. Thus, gelatin hydrolysate can be utilized to enhance rheological properties in low-fat ice cream instead of an emulsifier.

#### 2.5.1. Dynamic Rheological Properties

**Figure 2:**
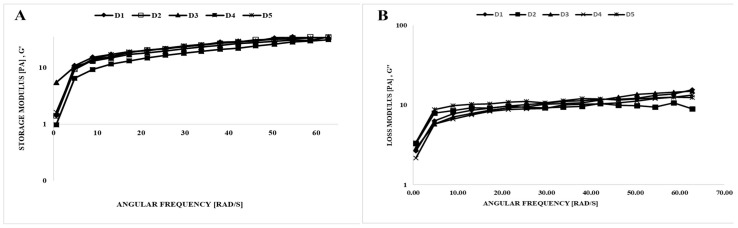
Dynamic rheological properties of the ice cream mixes. (**A**) storage modulus (*G*′) vs. ω, (**B**) loss modulus (*G*″) vs. ω.

Viscoelastic characteristics may be determined through dynamic rheological measurements, which facilitate the evaluation of structural evolution [24]. Figure 2 illustrates the use of the dynamic oscillatory shear test that was employed to define the viscoelastic characteristics of the mixes, determining storage (*G*′) and loss (*G*″) moduli. *G*′ values of all mixes surpassed *G*″ values, indicating greater elasticity compared to viscosity. The dynamic rheological properties of ice creams produced with different amounts of stabilizer, emulsifier, and gelatin hydrolysate were investigated. The quality of ice cream, as affected by the addition of varying amounts of stabilizer, emulsifier, and gelatin hydrolysate, can be comprehensively assessed using the frequency sweep test, which simulates the liquid behavior of the samples in the mouth during chewing [25]. Figure 2 indicates that polysaccharides increased the storage and loss modulus of ice cream mixes. The increasing *G*′ and *G*″ values of samples with the increasing frequency are evidence of gel-like behavior in ice cream samples [26]. In a study investigating the effect of incorporating okra gum into low-fat ice cream, Aziz et al. [27] noted that *G*′ values were greater than *G*″ values. The *G*′ and *G*″ values found in this study are in agreement with findings from previous research on viscoelastic properties [5,28]. Also, the D1 (sahlep and mono-diglyceride) and D3 (sahlep and gelatin hydrolysate) ice cream mixes had a similar storage modulus (Figure 2). Synergetic interactions between gelatin hydrolysate and stabilizers can contribute to enhanced food quality and advanced food applications.

The dynamic rheological parameters were also obtained by fitting the Power Law model (Table 3). High R^2^ (0.896–0.996) values indicated the accuracy of the fit of this model. The *K*′ and *K*″ values of the samples were in the range of 1.668–4.471 and 1.607–3.963, respectively; the values of n′ and n″ were found in the range of 0.463–0.667 and 0.126–0.278, respectively (Table 3). Due to the *K*′ values surpassing the *K*″ values, the ice cream mixes displayed viscoelastic solid behavior. Finally, these results were confirmed by previous studies on ice cream [5,28,29].

#### 2.5.2. Three Interval Thixotropy Test (3-ITT)

**Figure 3:**
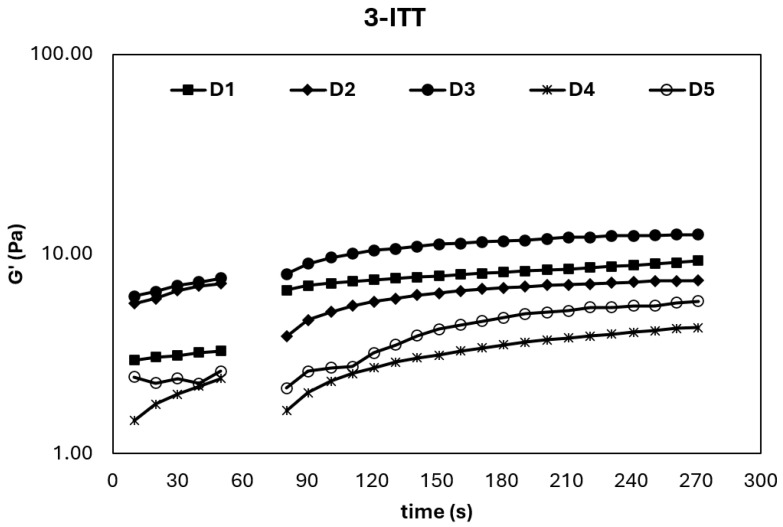
3-ITT rheological properties of ice cream mixes.

The 3-ITT provides critical insight into the structural breakdown and subsequent recovery behavior of ice cream mixes under sudden shear deformation, simulating industrial processes such as homogenization and pumping [5]. As shown in Figure 3, the storage modulus (*G*′) of all samples prominently increased in the third interval, confirming their thixotropic nature—a desirable feature for semi-solid food systems. This rise in *G*′ after shear cessation indicates that the viscoelastic network can rebuild itself, allowing the product to regain its structure and maintain texture during handling and storage [5].

These observations were further supported by the second-order structural kinetic model parameters summarized in Table 4, where the Ge/G0 ratio serves as an indicator of recovery efficiency. Among the samples, D1 and D3 exhibited the highest Ge/G0 and thixotropic rate constant (k) values, implying a faster and more complete structural recovery after breakdown. This suggests that the internal structure of these mixes is more resilient and capable of returning to its original state, which is vital for maintaining the quality and texture of ice cream throughout its lifecycle [30].

The enhanced thixotropic behavior, particularly in D1 (sahlep and mono-diglyceride) and D3 (sahlep and gelatin hydrolysate), may be attributed to the inclusion of gelatin hydrolysate–stabilizer complexes, which likely contribute to network restructuring and water-holding capacity. Similar findings were reported by Atik et al. [5], where cold-pressed chia seed oil by-products improved thixotropic performance. These results collectively indicate that the appropriate selection and combination of hydrocolloids and protein hydrolysates can be strategically used to optimize textural resilience, reduce phase separation, and improve the processing tolerance of ice cream mixes.

### 2.6. Sensory Properties

**Figure 4:**
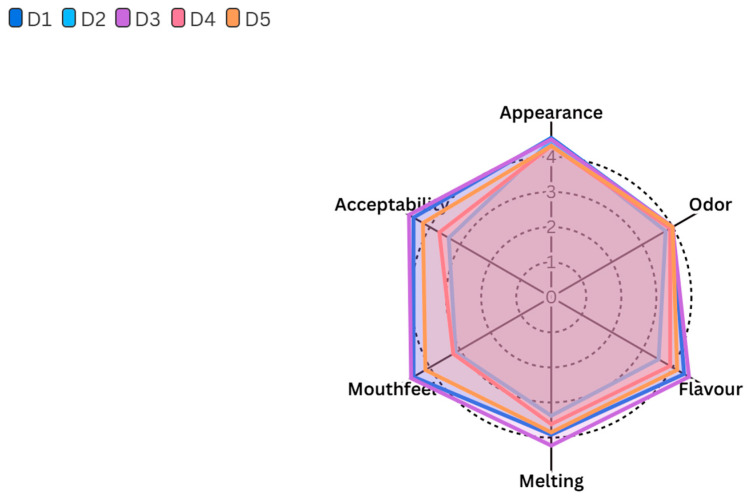
Sensory properties of ice cream samples.

The sensory properties of the ice cream samples, including appearance, odor, flavor, melting quality, mouthfeel, and overall acceptability, are presented in Figure 4. All ice cream samples scored comparably in terms of appearance, odor, flavor, and melting quality, indicating that the incorporation of gelatin hydrolysate, even as a complete substitute for stabilizers (D4 ice cream), did not adversely affect these sensory characteristics (*p* > 0.05). On the other hand, the mouthfeel scores of the ice cream samples were found statistically significant (*p* ≤ 0.05). The ice cream sample D3 (sahlep and gelatin hydrolysate) had the highest mouthfeel score, followed by the D1 (sahlep and mono-diglyceride) and D5 (sahlep, mono-diglyceride, and gelatin hydrolysate) ice cream samples, and the scores were statistically similar to each other. Conversely, ice cream samples D2 (mono-diglyceride and gelatin hydrolysate) and D4 (gelatin hydrolysate) received significantly lower scores for mouthfeel. This is particularly related to the absence of sahlep, which negatively affected the sensory characteristics of ice cream samples in terms of mouthfeel. Regarding overall acceptability scores of ice cream samples, there were also significant differences among them. The ice cream sample D3 received the highest acceptability score, and it was statistically higher than ice cream samples D2 and D4 (*p* ≤ 0.05). The control ice cream sample D1 and the D5 ice cream sample, containing equal amounts of sahlep, emulsifier, and gelatin hydrolysate, were not statistically different from the D3 ice cream sample (*p* > 0.05). As a result, the D2 (containing a high-level gelatin hydrolysate of an emulsifier) and D4 (containing solely gelatin hydrolysate) ice cream samples had the lowest acceptability scores. In conclusion, sensory scores of this study revealed that fish skin gelatin hydrolysate could be successfully used in ice cream production without negatively affecting appearance, odor, flavor, and melting quality. However, its effect on mouthfeel and overall acceptability is highly dependent on sahlep concentration. In this direction D3 ice cream, containing high concentration of sahlep and a low concentration of gelatin hydrolysate, had the most favorable sensory profile, particularly in terms of mouthfeel and general acceptability. In previous studies, the sensory effect of fish-based gelatin hydrolysate in various food products has been investigated. Chuaychan et al. [31] reported that gelatin hydrolysate derived from fish scales had no significant effect on the sensory acceptability of apple juice at concentrations up to 5%. Similarly, Kanwate et al. [32] reported that gelatin hydrolysate from rohu fish could be used in fish sausages at a level of 3–6% without changing their sensory properties. Furthermore, yogurt samples containing gelatin hydrolysate from sturgeon skin scored higher than the control yogurt sample in all sensory parameters except for color [33]. Finally, the findings obtained from this study and the literature data revealed that fish waste can be converted into gelatin hydrolysate or other derivatives and used for various purposes in food production processes.

## 3. Conclusions

This study demonstrated that gelatin hydrolysate derived from fish skin waste can be effectively utilized as a natural emulsifier and/or stabilizer in ice cream production. Its inclusion enhanced the protein content of the samples, improved rheological characteristics such as pseudoplastic behavior and viscoelasticity, and contributed to favorable structural recovery following deformation. Notably, ice cream formulations containing gelatin hydrolysate exhibited increased overrun levels and modified textural and melting properties, particularly in combinations with traditional stabilizers like sahlep. In particular, the ice cream formulation (D3), which used 0.25 g gelatin hydrolysate replacing the emulsifier in the presence of the traditional stabilizer, sahlep (0.75 g), achieved the highest sensory approval scores while maintaining the desired rheological and structural properties. While the use of gelatin hydrolysate alone reduced hardness and increased stickiness, its synergistic application with conventional agents yielded products with desirable firmness and resistance to melting. This suggests that gelatin hydrolysate can be used effectively, especially in synergy with traditional stabilizers. These findings highlight the potential of fish skin-derived gelatin hydrolysate as a sustainable and functional ingredient in ice cream production, offering both technological advantages and value-added utilization of seafood by-products.

## 4. Materials and Methods

### 4.1. Preparation of Gelatin Hydrolysate

The gelatin hydrolysate used as an emulsifier and/or stabilizer was obtained from sea bream (Sparus aurata). The production of gelatin and hydrolysate from the skins of sea bream was conducted in accordance with a published methodology [34]. Once the fish gelatin had been ground, a solution with a concentration of 5% (*w*/*v*) was made and dissolved to obtain a homogeneous solution. The pH of the Alcalase^®^ (3.042 U/mL, *Bacillus licheniformis*, Sigma-Aldrich Chemie GmbH, Taufkirchen, Germany) solution was adjusted to 8.0 at 60 °C using a 0.1 M NaOH solution. The enzyme was added at a 2.5% enzyme-to-substrate ratio, and hydrolysis was performed for 1 h. To terminate the hydrolysis and enzyme inactivation processes, the solutions were heated at 90 °C for 10 min, cooled to room temperature, and lyophilized by centrifugation at 10,000× *g* for 10 min (at 5 °C). The dry powdered hydrolysates were then packaged and stored (Hydrolysis degree: 16.16%).

### 4.2. Production of Ice Cream Samples

**Figure 5:**
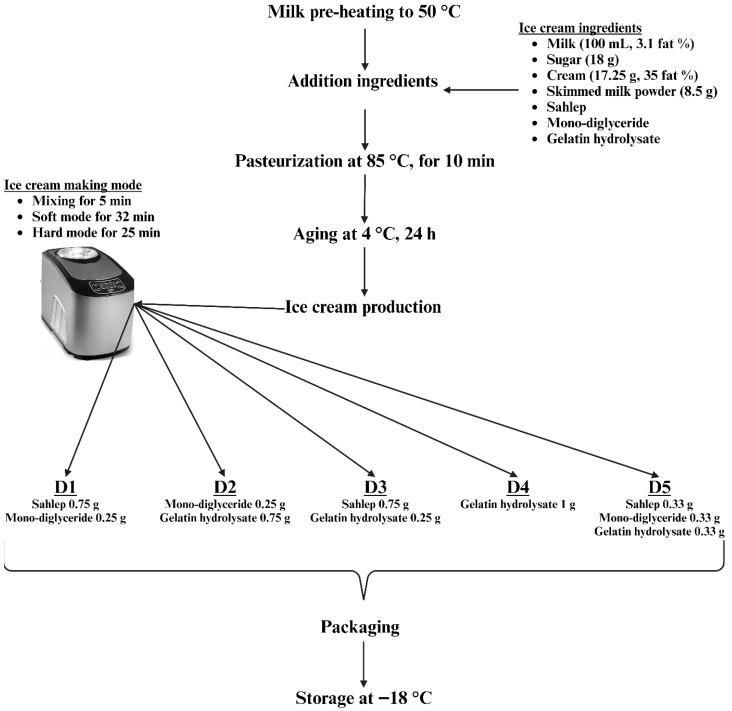
Ice cream production flow chart.

The recipe used in ice cream production was created in accordance with the specifications set forth by Goktas et al. [7]. To produce ice cream samples, since the fatty ice cream recipe was applied, the hard mode on the ice cream machine (ICM-15A, Vosco, Izmir, Turkey) was selected, and production was carried out as illustrated in Figure 5.

### 4.3. Physicochemical Properties

Melted ice cream samples were used to assess their pH, protein content, titratable acidity (TA), and total dry matter (TDM). pH values of the samples were recorded using a pH meter (Hanna HI2002-02, Hanna Instruments, Betuwehaven, Nieuwegein, The Netherlands). The TDM and TA analyses were performed following the procedures outlined by Williams [35]. Additionally, the Kjeldahl method was used to determine the protein content of the samples.

### 4.4. Color Properties

The color values of the samples were measured with a colorimeter (3nh NR200, Shenzhen, China) [36]. For each ice cream, two sets of L*, a*, and b* values were obtained, and the results were presented as the average along with the standard deviation.

### 4.5. Determination of Overrun Levels of Samples

The impact of incorporating fish skin gelatin hydrolysate as an emulsifier and/or stabilizer on the overrun level of ice cream was evaluated using the method outlined by Soukoulis et al. [37]. The overrun levels were calculated using the specified Equation (1):

(%)Overrun (%) = (Mix weight − Ice cream weight)/(Mix weight) × 100(1)

### 4.6. Textural Properties

A texture analyzer (TA-XT Express Stable Micro Systems) with a 10 kg load cell and a P/36R cylindrical aluminum probe was employed to assess the hardness and adhesiveness of the samples [36]. The hardness and adhesiveness values, respectively, were considered as the positive peak and negative peak forces (g) measured during the penetration of the probe into the sample. Before testing, the ice creams were cut into cubes and stored at −18 °C for 24 h. The texture analysis parameters were defined as follows: an initial speed of 2.00 mm/s, a test speed of 2.00 mm/s, and a final speed of 10.00 mm/s. A measurement distance of 40.00 mm and a force threshold of 0.1 N were also applied.

### 4.7. Melting Properties

To assess the MR and FDT of the ice creams, around 6 g of each sample was cut into cubes, rested at −18 °C for 2 h and held on a wire mesh with 2 mm pores at room temperature. The MR and FDT were recorded in minutes, then the total melted ice cream was weighed. Finally, MRs were determined using linear regression analysis and expressed as g/min [38].

### 4.8. Rheological Measurements

A rheometer (Anton Paar, Graz, Australia), temperature- and stress-controlled, was used to characterize the flow behavior, dynamic properties, and 3-ITT rheological properties of mixes. The measurements were carried out at 25 °C [28].

The flow behavior of mixes was identified at a shear rate from 0.1 to 100 (s^−1^) with a parallel plate probe (50 mm diameter and 0.5 mm gap size). The measurements were carried out in triplicate and at 25 °C. The data were fitted to the Power Law model, and the model parameters were determined using nonlinear regression.

τ = *K*γ*^n^* (2)

In Equation (2), the τ value represents the shear stress (Pa), *K* the consistency coefficient (Pa.s), γ the shear rate (s^−1^), and *n* the flow behavior index.

The linear viscoelastic region of the mixtures was first measured between 0.1% and 100% strain using a parallel plate configuration. Then, the frequency sweep test was performed between 0.1 and 10 Hz and 0.1 to 64 s^−1^(*ω*). The angular velocity and frequency values were used to calculate the elastic modulus (*G*′) and viscous modulus (*G*″). Nonlinear regression and the Power Law model were used to obtain the parameters for dynamic rheological characteristics.

*G*′ = *K*′(*ω*)^*n*′^ (3)

*G″* = *K″*(*ω*)^*n*″^ (4)

*G*′: storage modulus (Pa), *G*″: loss modulus (Pa), *ω*: angular velocity (s^−1^), *K*′ and *K*″: consistency coefficient values (Pa.s*^n^*), and *n*′ and *n*″: flow behavior index values.

The 3-ITT rheological qualities of the mixes were determined using a constant shear rate of 0.5 s^−1^ and a variable shear rate of 200 s^−1^. These values were chosen in accordance with the linear viscoelastic zone, which concludes at 10 s^−1^ for the samples. In the first time period, the ice cream samples were subjected to a very slow shear rate (0.5 s^−1^) for 50 s and a duration of 10 s. The specified cutting force was then applied at a rate of 200 s^−1^ for 20 s and a duration of 10 s during the second time period. The dynamic rheological behavior was examined in the third period (190.5 s and 0.5 s duration period) after they were exposed to the low shear rate in the first period. Throughout this process, the change in the viscoelastic solid structure (*G*′) of the samples was observed. To simulate the sample behavior in the third time period, a second-order structural kinetic model was utilized.

[(*G*′ − Ge)/(G0 − Ge)]^1−*n*^ = (*n* − 1)k × t − 1 (5)

*G*′: change in the storage modulus (Pa), G0: initial storage modulus value (Pa) in the third time interval, Ge: storage modulus at the point of the mixes’ complete recovery (Pa), k: thixotropic velocity constant.

### 4.9. Consumer Acceptance of the Ice Creams

The panelists were provided with the samples for the assessment of various sensory parameters, including appearance, odor, taste, melting quality, mouthfeel, and overall acceptability. The panel comprised 25 participants, consisting of 15 females and 10 males. Prior to the evaluation, the panelists received information regarding the study’s objectives, the criteria for scoring, and the specific sensory parameters under consideration. Each panelist was presented with approximately 25 g of samples in transparent plastic containers labeled with three randomly assigned numbers. During the evaluation process, panelists were instructed to rinse their mouths with water before sampling each ice cream variant. Subsequently, they were requested to rate each sample on a scale from 1 to 5 for each sensory attribute (1: never liked, 2: disliked, 3: acceptable, 4: liked, 5: very liked; for burnt flavor, the scale was defined as 1: intensely perceived, 2: slightly perceived, 3: perceived, 4: not perceived, 5: never perceived). The sensory evaluation form was designed following the methodology outlined by Soukoulis et al. [37].

### 4.10. Statistical Analyses

The data analysis for this study was performed using the Minitab statistical software v21.1.0. Results were reported as mean values along with their standard deviations. Statistical significance was assessed using Tukey’s test at a 95% confidence level.

## Figures and Tables

**Table 1:** Physicochemical and color properties of ice cream samples.

	Physicochemical Properties				Overrun (%)	Color Properties		
	pH	TA (%)	TDM (g/100 g)	Protein (g/100 g)		L*	a*	b*
D1	6.45 ± 0.00 ^a^	2.69 ± 0.09 ^a^	30.04 ± 0.06 ^a^	3.13 ± 0.07 ^b^	11.44 ± 1.79 ^cd^	87.41 ± 3.56 ^a^	−0.89 ± 0.16 ^ab^	10.43 ± 0.96 ^ab^
D2	6.46 ± 0.00 ^a^	2.73 ± 0.14 ^a^	30.04 ± 1.39 ^a^	3.75 ± 0.13 ^a^	9.55 ± 1.21 ^d^	86.67 ± 1.12 ^a^	−1.82 ± 0.33 ^d^	9.20 ± 1.55 ^b^
D3	6.48 ± 0.01 ^a^	2.75 ± 0.28 ^a^	30.01 ± 1.39 ^a^	3.32 ± 0.11 ^b^	21.74 ± 1.39 ^a^	89.67 ± 0.55 ^a^	−0.63 ± 0.09 ^a^	10.89 ± 0.91 ^a^
D4	6.48 ± 0.02 ^a^	2.80 ± 0.05 ^a^	29.97 ± 0.37 ^a^	3.99 ± 0.04 ^a^	15.79 ± 1.29 ^bc^	87.46 ± 2.29 ^a^	−1.47 ± 0.19 ^cd^	10.08 ± 0.61 ^ab^
D5	6.46 ± 0.00 ^a^	2.83 ± 0.18 ^a^	29.84 ± 0.25 ^a^	3.38 ± 0.03 ^b^	19.16 ± 1.60 ^ab^	88.51 ± 3.27 ^a^	−1.11 ± 0.13 ^bc^	9.92 ± 0.58 ^ab^

D1: sahlep (0.75 g) and mono-diglyceride (0.25 g), D2: mono-diglyceride (0.25 g) and gelatin hydrolysate (0.75 g), D3: sahlep (0.75 g) and gelatin hydrolysate (0.25 g), D4: gelatin hydrolysate (1 g), D5: sahlep (0.33 g), mono-diglyceride (0.33 g) and gelatin hydrolysate (0.33 g). TA: titratable acidity, TDM: total dry matter. Levels not connected by same letter are significantly different (*p* ≤ 0.05).

**Table 2:** Steady shear Power Law parameters, textural and melting properties of the ice cream samples.

	Steady Shear Properties			Textural Properties		Melting Properties	
	K (Pa.s)	*n*	R^2^	Hardness (g)	Adhesiveness (g)	FDT (min)	MR (g/min)
D1	1.012 ± 0.012 ^a^	0.558 ± 0.004 ^c^	1.000 ± 0.000	1511.59 ± 302.78 ^bc^	−357.66 ± 62.69 ^bc^	3.74 ± 0.56 ^b^	0.62 ± 0.05 ^a^
D2	0.249 ± 0.033 ^b^	0.356 ± 0.019 ^d^	0.989 ± 0.016	1703.51 ± 267.03 ^b^	−403.61 ± 84.36 ^c^	5.23 ± 0.18 ^ab^	0.52 ± 0.01 ^ab^
D3	0.997 ± 0.064 ^a^	0.550 ± 0.009 ^c^	1.000 ± 0.000	1597.32 ± 653.19 ^bc^	−248.85 ± 25.31 ^b^	5.21 ± 0.09 ^ab^	0.57 ± 0.00 ^ab^
D4	0.009 ± 0.003 ^c^	0.863 ± 0.026 ^a^	0.996 ± 0.003	803.19 ± 141.76 ^c^	−92.05 ± 41.45 ^a^	6.67 ± 0.63 ^a^	0.53 ± 0.01 ^ab^
D5	0.190 ± 0.047 ^b^	0.664 ± 0.052 ^b^	1.000 ± 0.000	2826.95 ± 451.29 ^a^	−606.35 ± 98.93 ^d^	5.52 ± 0.07 ^a^	0.49 ± 0.04 ^b^

D1: sahlep (0.75 g) and mono-diglyceride (0.25 g), D2: mono-diglyceride (0.25 g) and gelatin hydrolysate (0.75 g), D3: sahlep (0.75 g) and gelatin hydrolysate (0.25 g), D4: gelatin hydrolysate (1 g), D5: sahlep (0.33 g), mono-diglyceride (0.33 g) and gelatin hydrolysate (0.33 g). FDT: first dripping time, MR: melting rate. Levels not connected by same letter are significantly different (*p* ≤ 0.05).

**Table 3:** Dynamical rheological parameters of ice cream mixes.

	*K*′	*n*′	R^2^	*K*″	*n*″	R^2^
D1	4.457 ± 0.169 ^a^	0.519 ± 0.008 ^c^	0.996 ± 0.004	3.481 ± 0.020 ^b^	0.272 ± 0.086 ^a^	0.975 ± 0.009
D2	2.124 ± 0.566 ^c^	0.601 ± 0.018 ^b^	0.997 ± 0.001	1.774 ± 0.133 ^d^	0.126 ± 0.024 ^a^	0.981 ± 0.012
D3	4.471 ± 0.284 ^a^	0.492 ± 0.005 ^cd^	0.994 ± 0.004	3.963 ± 1.623 ^a^	0.278 ± 0.161 ^a^	0.941 ± 0.064
D4	1.668 ± 0.431 ^c^	0.667 ± 0.011 ^a^	0.995 ± 0.003	1.607 ± 0.025 ^d^	0.243 ± 0.136 ^a^	0.896 ± 0.130
D5	3.783 ± 1.043 ^b^	0.463 ± 0.043 ^d^	0.974 ± 0.032	2.573 ± 0.422 ^c^	0.271 ± 0.118 ^a^	0.947 ± 0.001

D1: sahlep (0.75 g) and mono-diglyceride (0.25 g), D2: mono-diglyceride (0.25 g) and gelatin hydrolysate (0.75 g), D3: sahlep (0.75 g) and gelatin hydrolysate (0.25 g), D4: gelatin hydrolysate (1 g), D5: sahlep (0.33 g), mono-diglyceride (0.33 g) and gelatin hydrolysate (0.33 g). Levels not connected by same letter are significantly different (*p* ≤ 0.05).

**Table 4:** 3-ITT rheological parameters of ice cream mixes.

	G0	Ge	k	Ge/G0	*K* × 1000	R^2^
D1	1.437 ± 0.041 ^b^	6.673 ± 0.412 ^b^	0.022 ± 0.002 ^a^	4.639 ± 0.156 ^a^	22.197 ± 0.226 ^a^	0.999 ± 0.000
D2	2.997 ± 0.041 ^b^	8.624 ± 0.251 ^b^	0.005 ± 0.000 ^bc^	2.877 ± 0.044 ^b^	4.934 ± 0.154 ^d^	1.000 ± 0.000
D3	2.077 ± 0.121 ^b^	8.658 ± 0.136 ^b^	0.022 ± 0.005 ^a^	4.179 ± 0.177 ^a^	21.523 ± 0.247 ^ab^	0.996 ± 0.002
D4	6.748 ± 0.132 ^a^	13.832 ± 0.104 ^a^	0.002 ± 0.000 ^bc^	2.050 ± 0.025 ^c^	1.912 ± 0.289 ^e^	0.997 ± 0.001
D5	6.399 ± 0.206 ^a^	14.018 ± 0.708 ^a^	0.008 ± 0.001 ^b^	2.189 ± 0.040 ^c^	7.682 ± 0.623 ^c^	0.982 ± 0.017

D1: sahlep (0.75 g) and mono-diglyceride (0.25 g), D2: mono-diglyceride (0.25 g) and gelatin hydrolysate (0.75 g), D3: sahlep (0.75 g) and gelatin hydrolysate (0.25 g), D4: gelatin hydrolysate (1 g), D5: sahlep (0.33 g), mono-diglyceride (0.33 g) and gelatin hydrolysate (0.33 g). Levels not connected by same letter are significantly different (*p* ≤ 0.05).

## Data Availability

The data that assisted the results of this work can be provided from the corresponding writer upon acceptable demand.

## Abbreviations

The following abbreviations are used in this manuscript:

FDT	First dripping time
MR	Melting rate
TA	Titratable acidity
TDM	Total dry matter
